# Poly(Glutamic Acid‐Lysine) Hydrogels with Alternating Sequence Resist the Foreign Body Response in Rodents and Non‐Human Primates

**DOI:** 10.1002/advs.202308077

**Published:** 2024-02-25

**Authors:** Xianchi Zhou, Wenzhong Cao, Yongcheng Chen, Zihao Zhu, Yifeng Chen, Yanwen Ni, Zuolong Liu, Fan Jia, Zhouyu Lu, Yang Ye, Haijie Han, Ke Yao, Weifeng Liu, Xinyue Wei, Shengfu Chen, Youxiang Wang, Jian Ji, Peng Zhang

**Affiliations:** ^1^ MOE Key Laboratory of Macromolecule Synthesis and Functionalization of Ministry of Education Department of Polymer Science and Engineering Zhejiang University Hangzhou Zhejiang 310058 P. R. China; ^2^ Key Laboratory of Cardiovascular Intervention and Regenerative Medicine of Zhejiang Province Department of Cardiology Sir Run Run Shaw Hospital School of Medicine Zhejiang University Hangzhou Zhejiang 310016 P. R. China; ^3^ Eye Center The Second Affiliated Hospital School of Medicine Zhejiang Provincial Key Laboratory of Ophthalmology Zhejiang Provincial Clinical Research Center for Eye Diseases Zhejiang Provincial Engineering Institute on Eye Diseases Zhejiang University Hangzhou Zhejiang 310009 P. R. China; ^4^ Department of Hepatobiliary and Pancreatic Surgery The Second Affiliated Hospital School of Medicine Zhejiang University Hangzhou Zhejiang 310009 P. R. China; ^5^ Key Laboratory of Biomass Chemical Engineering of Ministry of Education Department of Chemical and Biological Engineering Zhejiang University Hangzhou Zhejiang 310058 P. R. China; ^6^ International Research Center for X Polymers International Campus Zhejiang University Haining Zhejiang 314400 P. R. China; ^7^ State Key Laboratory of Transvascular Implantation Devices Zhejiang University Hangzhou Zhejiang 311202 P. R. China

**Keywords:** biodegradable, foreign body response, implant, inflammation, polypeptide hydrogel

## Abstract

The foreign body response (FBR) to implanted biomaterials and biomedical devices can severely impede their functionality and even lead to failure. The discovery of effective anti‐FBR materials remains a formidable challenge. Inspire by the enrichment of glutamic acid (E) and lysine (K) residues on human protein surfaces, a class of zwitterionic polypeptide (ZIP) hydrogels with alternating E and K sequences to mitigate the FBR is prepared. When subcutaneously implanted, the ZIP hydrogels caused minimal inflammation after 2 weeks and no obvious collagen capsulation after 6 months in mice. Importantly, these hydrogels effectively resisted the FBR in non‐human primate models for at least 2 months. In addition, the enzymatic degradability of the gel can be controlled by adjusting the crosslinking degree or the optical isomerism of amino acid monomers. The long‐term FBR resistance and controlled degradability of ZIP hydrogels open up new possibilities for a broad range of biomedical applications.

## Graphical abstract

Inspired by the enrichment of glutamic acid (E) and lysine (K) residues on human protein surfaces, a class of zwitterionic polypeptide (ZIP) hydrogels with alternating E and K sequences to mitigate the FBR is prepared. When subcutaneously implanted, the ZIP hydrogels causes minimal inflammation and no obvious collagen capsulation in mice and non‐human primates.

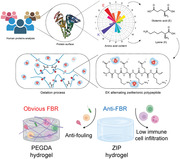

## 1 Introduction

Implantable biomedical devices and biomaterials occupy a pivotal role in the modern medical field, encompassing an array of applications such as breast implants,^[^ ^1^ ^]^ neural implants,^[^ ^2^ ^]^ pacemakers,^[^ ^3^ ^]^ and devices for cell encapsulation and transplantation.^[^ ^4^, ^5^, ^6^ ^]^ However, the foreign body response (FBR) triggered by these implanted materials can lead to fibrotic encapsulation, compromising the efficacy of implants and giving rise to complications.^[^ ^7^, ^8^, ^9^, ^10^, ^11^, ^12^, ^13^ ^]^ The FBR can be elicited by a wide range of materials employed in the fabrication of biomedical devices, irrespective of whether they are composed of metals, inorganics, or natural and synthetic polymers.^[^ ^14^, ^15^, ^16^, ^17^ ^]^ Surprisingly, even materials conventionally known for their favorable biocompatibility, such as hydrophilic polymers like polyethylene glycol (PEG) and poly(2‐hydroxyethyl methacrylate), have been found to evoke strong FBR.^[^ ^14^, ^15^, ^18^ ^]^ The last decade has witnessed the emergence of pure zwitterion hydrogels adept at thwarting nonspecific protein adsorption and substantially alleviating the FBR.^[^ ^18^, ^19^ ^]^ However, these zwitterionic hydrogels are not metabolically biodegradable, raising concerns over potential surgical removal that may lead to patient harm and additional expenses. Although the inclusion of degradable crosslinkers can make those hydrogels degradable, the high molecular weight polymer residues will remain and accumulate in the body after the degradation of those hydrogels. In specific in vivo biomedical applications such as drug delivery,^[^ ^20^ ^]^ disposable catheters, and materials for preventing postoperative adhesion,^[^ ^21^, ^22^ ^]^ ultra‐long‐term implantation may not be imperative. Hence, a compelling demand exists for a safe biomaterial capable of controlled in vivo degradation while concurrently exhibiting long‐term resistance to the FBR.

EK polypeptide materials originated from the analysis of the human protein database. An intriguing observation is the large fractions of lysine (K) and glutamic acid (E) residuals on the human protein surface, which possess robust water‐binding capabilities but weak binding with surrounding amino acids.^[^ ^23^, ^24^ ^]^ The pseudo zwitterionic polypeptide material, synthesized through alternating copolymerization of glutamic acid and lysine or aspartic acid and lysine,^[^ ^25^, ^26^, ^27^, ^28^, ^29^ ^]^ has demonstrated enzymatic degradability while retaining its inherent zwitterionic characteristics to resist nonspecific protein adsorption.^[^ ^17^, ^26^, ^30^, ^31^, ^32^, ^33^ ^]^ Furthermore, the degradation products consist of naturally occurring amino acids that can undergo metabolic processes within the natural protein metabolic pathway. Although poly(EK) has been applied for surface coatings,^[^ ^26^, ^34^ ^]^ protein conjugation,^[^ ^35^, ^36^ ^]^ and drug delivery,^[^ ^37^, ^38^, ^39^ ^]^ the study of poly(EK) hydrogels remains scarce due to the difficulties associated with the synthesis of the alternating polypeptides. Despite there having been attempts to develop EK hydrogels, the process has relied on N‐carboxyanhydride (NCA) ring‐opening random polymerization, resulting in hydrogels lacking a precisely defined zwitterionic alternating structure.^[^ ^31^, ^32^ ^]^ Additionally, the uncontrolled rapid in vivo degradation of EK materials poses a significant limitation to their broader application. Despite the well‐established recognition of the impressive anti‐fouling properties of EK polypeptide materials, no direct evidence indicates their ability to alleviate the FBR associated with implants.^[^ ^32^, ^40^, ^41^ ^]^

Here, we have developed a series of ZIP hydrogels with a well‐defined alternating copolymerization pattern of glutamic acid and lysine inspired by the observation of a notable abundance of E and K amino acids on human protein surfaces. This ZIP hydrogel can undergo complete degradation into naturally occurring amino acids within the human body. While polypeptides made from L‐type amino acids undergo relatively fast degradation in vivo, undegradable D‐type amino acids can be added into ZIP hydrogels to adjust the degradation kinetics. The ZIP hydrogel exhibits exceptional resistance to protein adsorption, cellular adhesion, and bacterial colonization in vitro. Moreover, the hydrogel elicits negligible inflammation and effectively resists the FBR in both murine and non‐human primate (NHP) models for 6 months and 2 months, respectively. To the best of our knowledge, this marks the initial successful synthesis of a zwitterionic polypeptide hydrogel with an alternating sequence. The ZIP hydrogel is entirely biodegradable, meaning that the main chain of the polymer can be biologically metabolized into amino acids. The degradability together with the long‐term fibrosis resistance indicates its potential for implantable devices.

## 2 Result

### 2.1 Biofouling and Enzymatic Degradation Test of ZIP Hydrogels

**Figure 1:**
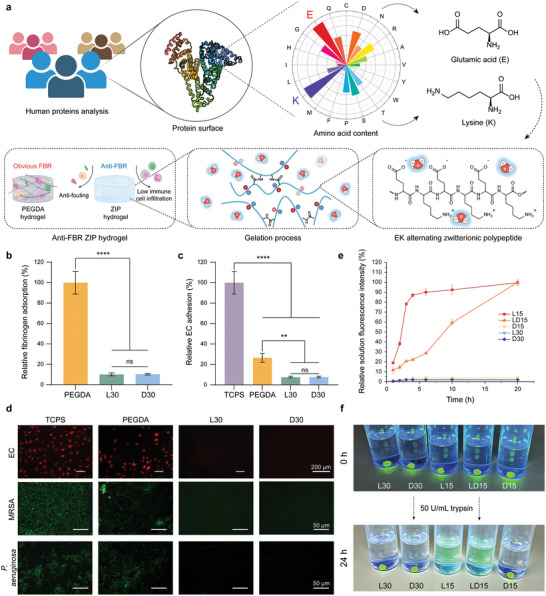
Design and in vitro tests of ZIP hydrogels. a) Schematic diagram of origin, structure, and function of ZIP hydrogels. b) Quantitative measurements of fibrinogen adsorption (data were normalized to that of PEGDA sample, *n* = 6 hydrogels per group, mean ± s.d.). c) The quantitative measurements of EC adhesion (data were normalized to that of TCPS sample, *n* = 4 hydrogels per group, mean ± s.d.). d) Microscopy images for EC adhesion and bacteria attachment. e) The quantitative measurements of fluorescence signal intensity of FITC in solution (*n* = 3 hydrogels per group, mean ± s.d.). f) photographs of FITC‐labeled ZIP hydrogels before and after 24 h of trypsin treatment. Statistical significance was determined by one‐way analysis of variance (ANOVA) with Turkey post‐test. ^*^ *p* < 0.05, ^**^ *p* < 0.01, ^***^ *p* < 0.001, ^****^ *p* < 0.0001, ns: not significant.

In our previous work, we devised an EK dimer strategy for synthesizing charge‐alternating polypeptides, successfully achieving zwitterionic polypeptide with a well‐defined alternating structure (Figure S1 and Table S1, Supporting Information).^[^ ^27^ ^]^ Compared with traditional peptide solid‐phase synthesis techniques, this method enables the cost‐effective and large‐scale production of alternating EK peptides. In this work, we made attempts to convert zwitterionic polypeptide materials into hydrogels. To preserve the inherent zwitterionic structural attributes of the EK alternating peptide hydrogel to the greatest possible extent, we employed *N*‐(3‐Dimethylaminopropyl)‐*N*'‐ethylcarbodiimide hydrochloride (EDCl) as an activation agent to reactivate carboxyl groups and facilitate their crosslinking reaction with the amino groups present within the polypeptide (**Figure** **1**a). The ZIP hydrogels with varying crosslinking degrees were fabricated utilizing the EK polypeptide derived from amino acid monomers with different configurations (Figure S2 and Table S2, Supporting Information). The ZIP hydrogels with varying degrees of cross‐linking exhibit distinct swelling and rheological properties (Figures S3 and S4, Supporting Information). The incorporation of D‐type amino acids in the peptide design is motivated by the inherent protease degradation resistance of D‐peptides.^[^ ^42^ ^]^ ZIP hydrogels were designated based on the amino acid monomer configuration followed by the crosslinker EDCl feeding ratio. For instance, a hydrogel prepared using L‐type amino acids and an EDCl feeding ratio of 30% molar ratio of amino groups on peptides was denoted as L30. Considering that nonspecific protein adsorption serves as the initial stage of the FBR, we employed fibrinogen (Fg, 340 kDa, pI 5.5), a large blood plasma protein known for its strong affinity toward hydrophobic surfaces, to evaluate the protein adsorption levels of the ZIP materials. Surface fibrinogen adsorption was quantified using an enzyme‐linked immunosorbent assay (ELISA) method (Figure 1b). Both the L30 and D30 hydrogels demonstrated remarkable non‐fouling capabilities, significantly reducing fibrinogen adsorption by 89.9% and 89.7%, respectively, in comparison to PEGDA hydrogels. The assessment of endothelial cell (EC) adhesion was conducted using a fluorescence staining assay and a cell counting kit‐8 (CCK‐8) assay (Figure 1c,d). Minimal EC adhesion was observed on both the L30 and D30 hydrogels at 4 h post‐cell seeding. In stark contrast, substantial EC adhesion with the extension of thin pseudopodia was observed on tissue culture polystyrene (TCPS) and poly(ethylene glycol) diacrylate (PEGDA) hydrogel surfaces. Quantitative analysis disclosed that the L30 and D30 hydrogels impressively suppressed EC attachment by approximately 92.4% compared to TCPS. Additionally, the ZIP hydrogels also exhibited robust resistance to bacterial attachment. We assessed the attachment of a Gram‐positive MRSA strain and a Gram‐negative *P. aeruginosa* strain, both being primary pathogens responsible for infections in healthcare settings and predominant colonizers on various biomedical implants. Minimal bacterial presence was observed on the surfaces of L30 and D30 hydrogels following bacterial incubation, with scant bacteria detected. In contrast, significant bacterial attachment and colonization were noted on TCPS and PEGDA hydrogel surfaces (Figure 1d).

We continued to evaluate the degradation behavior of ZIP hydrogels with different crosslinking degrees and amino acid monomer configurations through in vitro trypsin degradation experiments. FITC‐labelled ZIP hydrogels were subjected to trypsin‐induced degradation conditions. The results revealed a rapid degradation trend for L15 gels with low crosslinking degree and L‐type monomer configuration. Interestingly, the degradation pace of LD15 gels exhibited a notable deceleration subsequent to the incorporation of half of D‐type amino acids, all while maintaining an equivalent crosslinking degree. The remaining hydrogels with a high crosslinking degree or D configuration showcased negligible degradation after a 20‐h trypsin exposure (Figure 1e,f).

### 2.2 In Vivo Degradation Behavior and Antifibrotic Capabilities of ZIP Hydrogels

**Figure 2:**
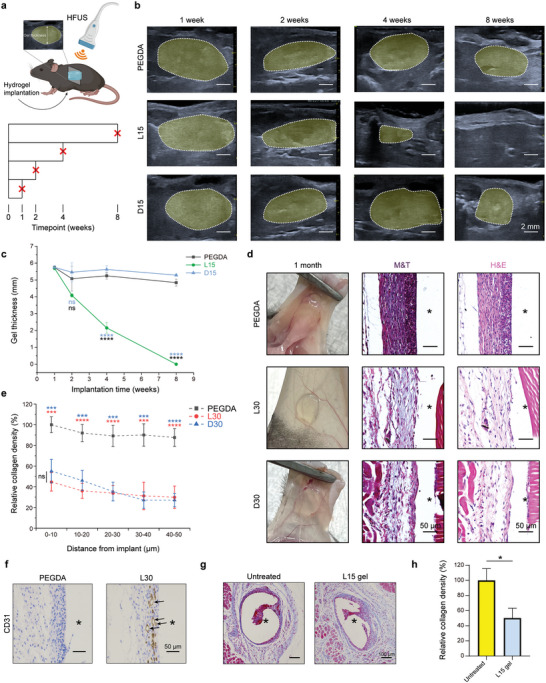
Degradation kinetics and in vivo FBR evaluation of ZIP hydrogels. a–c) Discs made of PEGDA and ZIP hydrogels (L15, L30, D15, and D30) with a diameter of 4 mm and a thickness of 1 mm were implanted subcutaneously (SubQ) on mouse backs. HFUS imaging evaluated changes in hydrogel thickness over time (HFUS images in b and quantitative thickness data in c) (*n* = 3 mice per group, mean ± s.d.). d) Digital photos, H&E‐, and Masson's trichrome (M&T)‐stained histological sections of excised tissue 1‐month post‐implantation. Asterisks in the section images denote the original locations of the implants. e) Density of the collagen capsule of the hydrogel‐tissue interface after 4 weeks of implantation (*n* = 4 mice per group, mean ± s.d.). f) Representative blood vessel staining images in C57BL/6 mice. Blood vessel staining (brown) using CD31 antibody on tissues surrounding SC implanted hydrogels from different groups at day 14 post‐implantation in healthy C57BL/6 mice. The arrow indicates newly formed blood vessels around the ZIP hydrogel. (*n* = 3 mice). g) Representative Masson trichrome‐stained images of tissues surrounding SC‐implanted catheters from different groups at day 14 post‐implantation. (*n* = 3 mice, mean values ± s.d.). h) Density of the collagen capsule of the catheter‐tissue interface. Statistical significance was determined by one‐way analysis of variance (ANOVA) with Turkey post‐test and unpaired, two‐tailed t‐test (h). ^*^ *p* < 0.05, ^**^ *p* < 0.01, ^***^ *p* < 0.001, ^****^ *p* < 0.0001, ns: not significant.

Next, our investigation extended to subcutaneous implantation experiments in mice to investigate the in vivo degradation behavior of ZIP hydrogels over time. The degradation process was monitored by assessing changes in hydrogel thickness using high‐frequency ultrasound (HFUS) imaging (**Figure** **2**a). Notably, L15 gels exhibited significant degradation by the 4‐week postimplantation mark, ultimately disappearing by 8 weeks. This phenomenon was confirmed by both HFUS imaging and visual observations upon sample retrieval (Figure 2b; Figure S5, Supporting Information). In contrast, the D15 and PEGDA control gels did not show obvious degradation (Figure 2c). Notably, the L30 and D30 gels with high crosslinking degrees, demonstrated no discernible signs of degradation until the 16‐week mark, as confirmed by HFUS imaging (Figure S6, Supporting Information). In conclusion, elevating the crosslinking degree or integrating D‐type amino acids emerged as effective strategies for enhancing the ZIP hydrogel's resistance against enzymatic hydrolysis and vice versa.

For further in vivo tests, we selected ZIP hydrogels with high crosslinking degrees, specifically L30 and D30, due to their demonstrated long‐term in vivo stability. One of the most commonly used materials for biomedical applications, the PEGDA hydrogel was used for comparison for the following implantation studies. It is well‐acknowledged that physical properties, including modulus,^[^ ^43^ ^]^ size, and shape of the specimen can impact the magnitude of the fibrotic response.^[^ ^44^ ^]^ To minimize the potential influences, we harmonized the modulus of PEGDA control samples (∼0.07 MPa), which is slightly lower than L30 and D30 (∼0.11 MPa, Figure S7, Supporting Information). The subcutaneous implantation tests were conducted on C57BL/6 mice with each mouse receiving implants of all three samples on its back (Figure S8, Supporting Information). The discs were retrieved one month after implantation. Representative photographs and histological staining (Masson's trichrome and haematoxylin and eosin (H&E)) revealed that the PEGDA hydrogels were encapsulated by a thick and dense fibrotic capsule, while ZIP hydrogels were ensconced in a more loosely arranged collagen matrix (Figure 2d). Further quantification via collagen density analysis substantiated these observations, indicating a ∼50% collagen density for ZIP hydrogels as compared to PEGDA hydrogels. This stark difference underscores the diminished FBR provoked by ZIP hydrogels (Figure 2e). Zwitterionic hydrogels have been reported to have pro‐angiogenic abilities.^[^ ^18^, ^45^ ^]^ In our work, the immunohistochemical analysis using CD31 staining demonstrated a notably increased density of blood vessels in the tissue adjacent to the L30 hydrogels compared to the tissue surrounding the PEG hydrogels (Figure 2f). The elevated presence of blood vessels around ZIP hydrogels, in contrast to PEG hydrogels, implies a more favorable milieu for crucial substance exchanges, such as the efficient transport of nutrients and oxygen around the implanted ZIP hydrogels. These attributes are particularly crucial for medical devices involved in drug and cell delivery, such as insulin infusion catheters.^[^ ^46^ ^]^ To demonstrate the practical utility of our method in medical devices, we enveloped the insulin infusion catheter with preinjected ZIP hydrogels and assessed the fibrotic response 2 weeks postimplantation in mice. The results show that ZIP hydrogels can significantly reduce the collagen density around the catheter compared with untreated groups, indicating the potential of the ZIP hydrogel to improve insulin pump performance in vivo (Figure 2g,h).

### 2.3 ZIP Hydrogels Elicited a Negligible Acute Inflammatory Response

**Figure 3:**
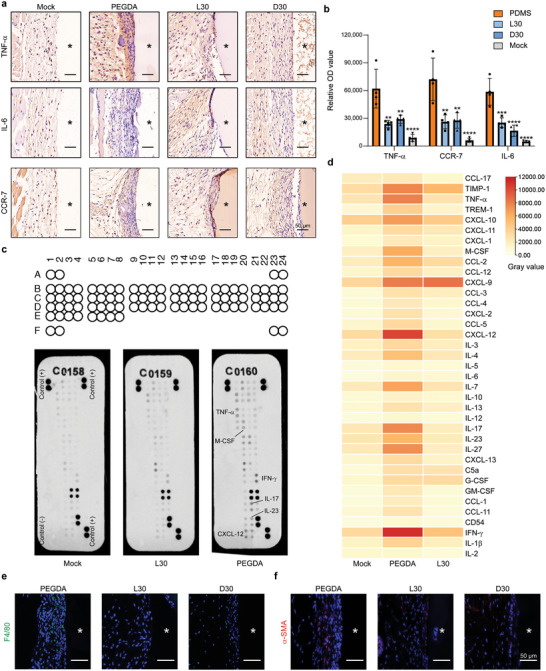
Inflammatory marker staining and cytokine profiling analysis 2‐week postimplantation. a,b) Discs made of PEGDA and ZIP hydrogels (L30 and D30) with a diameter of 4 mm and a thickness of 1 mm were implanted SubQ on mouse backs. The tissue surrounding the implant was analyzed 14 days postimplantation to evaluate the acute inflammation. Immunohistochemistry (IHC) was performed for inflammatory markers (TNF‐α, IL‐6, and CCR7) with staining shown in (a) and quantification shown in (b). Cells stained by inflammatory markers show a brown color, while all nuclei stained with hematoxylin show a blue color. Data were collected in the tissue within 50 µm from the tissue‐material interface (*n* = 3 mice per group, mean ± s.d.). c) Cytokine profiling for tissue lysates 2 weeks postimplantation of PEGDA and ZIP hydrogels, using mock group without implantation as control. The mouse cytokine array coordinates are shown in Table S3 (Supporting Information). d) Gray values of cytokine and chemokine expression. e, f) Immunofluorescence staining densities of F4/80 (green) (e) and α‐SMA (red) (f). Counterstaining was performed with DAPI (blue) for cell nuclei. Statistical significance was determined by an unpaired, two‐tailed t‐test. ^*^ *p* < 0.05, ^**^ *p* < 0.01, ^***^ *p* < 0.001, ^****^ *p* < 0.0001, ns: not significant.

Subsequently, we undertook an investigation to assess the acute inflammatory response triggered by ZIP hydrogels upon subcutaneous implantation in C57BL/6 mice for a duration of two weeks. Remarkably, both L30 and D30 hydrogels exhibited slight signs of inflammation. To examine the impact of hydrogel implantation on local tissue inflammation, we performed immunohistochemical sectioning to target key pro‐inflammatory markers, including TNF‐α, IL‐6, and CCR‐7. The results revealed a significant increase in inflammatory markers at the interface between the tissue and PEGDA hydrogel. In contrast, L30 and D30 gels exhibited considerably lower expression of inflammatory markers, effectively reducing the levels to ∼1/2 to 1/4 of those observed around the PEGDA hydrogel (**Figure** **3**a,b). Next, to comprehensively evaluate the inflammatory response induced by the implanted ZIP hydrogels, we utilized proteome profiler antibody arrays to analyze multiple inflammation‐related cytokines and chemokines in the tissues adjacent to the implants after two weeks of implantation. The mock group, unsubjected to implantation, served as a reference. The outcomes unveiled significantly reduced levels of several chemokines, encompassing C‐C motif chemokine ligand 2 (CCL‐2), C‐X‐C motif chemokine ligand 10 (CXCL‐10), and C‐X‐C motif chemokine ligand 12 (CXCL‐12); inflammatory cytokines, including interleukin‐7 (IL‐7), interleukin‐17 (IL‐17), and interleukin‐27 (IL‐27); interferon‐gamma; and colony‐stimulating factors in the tissues surrounding the ZIP hydrogel implants compared with PEGDA implants (Figure 3c,d; Table S3, Supporting Information).

In pursuit of a more profound comprehension of the cellular components driving the fibrotic response around ZIP hydrogels, we examined the recruitment of pivotal FBR‐related cells at the tissue‐hydrogel interface 2 weeks after subcutaneous implantation (Figure 3e,f). Immunofluorescence staining for myofibroblasts (α‐smooth muscle actin (α‐SMA)) and macrophages (F4/80) unveiled the substantial influx of fibroblasts and macrophages toward the PEGDA‐tissue interface, whereas ZIP gels exhibited a notably restrained degree of cellular overgrowth.

### 2.4 Less Profibrotic Inflammation was Induced by ZIP Hydrogels 1‐Month Post‐implantation

**Figure 4:**
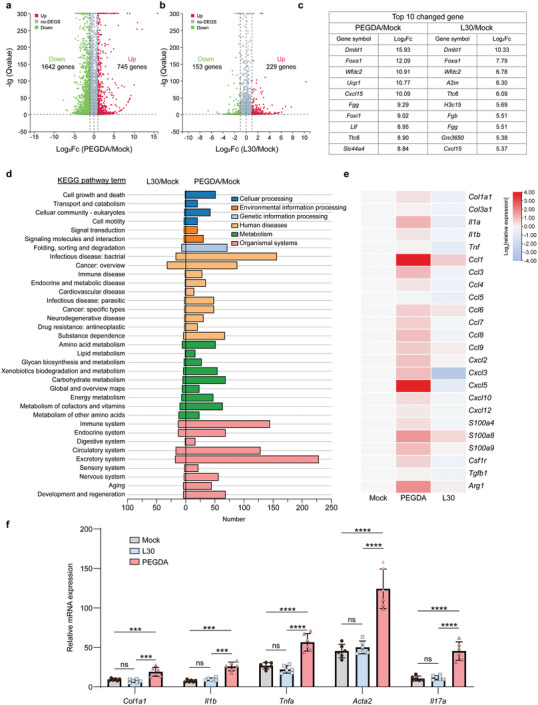
RNA‐seq and q‐PCR test 1‐month post‐implantation. a–c) Significant gene expression changes of PEGDA/mock comparison (a) and slight changes of L30/mock comparison (b) in genes, and a list of top 10 upregulated genes and corresponding log_2_Fc (log_2_ fold change) (c). d) KEGG pathway classification analysis of PEGDA/mock comparison and L30/mock comparison. e) Gene expression analysis of inflammatory factors in tissues surrounding the PEGDA and L30, with data normalized to the mock group (*n* = 3 mice per group). f) qRT‐PCR analysis of mRNA expression for FBR‐related genes (*n* = 6 mice per group, mean ± s.d.). Statistical significance was determined by one‐way analysis of variance (ANOVA) with Turkey post‐test. ^*^ *p* < 0.05, ^**^ *p* < 0.01, ^***^ *p* < 0.001, ^****^ *p* < 0.0001, ns: not significant.

We conducted RNA‐sequencing (RNA‐seq) analysis on tissues adjacent to the hydrogels 1‐month post‐implantation, utilizing a mock group without implantation as the control (**Figure** **4**a–e). Applying a significance threshold of *p* value < 0.05, we identified 2387 differentially expressed genes in the PEGDA and mock groups, whereas only 382 genes displayed differential expression in the L30 and mock groups (Figure 4a,b), indicating a minor immune reaction induced by the L30 hydrogels. Among the top 10 upregulated genes listed in the table, we observed higher overall expression levels of upregulated genes in response to PEGDA hydrogels compared to L30 hydrogels (Figure 4c). For a more comprehensive insight, we delved into KEGG pathway classification analysis and observed that the PEGDA/mock comparison generally had a higher number of differential genes than the L30/mock comparison, within which we found 88 and 32 differential genes respectively for the PEGDA/mock comparison and L30/mock comparison in the immune system (Figure 4d). Notably, the L30 hydrogel alleviated the RNA expression of the FBR‐related factors colony‐stimulating factor 1 receptor (Csf1r) and transforming growth factor beta 1 (Tgfb1); fibrosis markers collagen, type I, alpha 1 (Col1a1) and collagen, type III, alpha 1 (Col3a1); inflammatory markers tumor necrosis factor, interleukin 1 alpha (Il1a), and interleukin 1 beta (Il1b); chemokines C‐C motif chemokine ligand 1 (Ccl1) and C‐X‐C motif chemokine ligand 5 (Cxcl5); and inflammatory response‐related proteins S100 calcium binding protein A8 (S100a8) and S100 calcium binding protein A9 (S100a9) compared with PEGDA implants (Figure 4e).

To double‐check the key factors associated with the FBR, we performed a quantitative polymerase chain reaction (qPCR) analysis one month after implantation using ZIP hydrogels (Figure 4f; Table S4, Supporting Information). FBR‐associated gene expressions did not exhibit significant differences between the tissues surrounding L30 and normal tissues, including interleukin 6 (Il6), interleukin 1 beta, actin alpha 2 (Acta2), colony‐stimulating factor 1 (Csf1), C‐X‐C motif chemokine ligand 13 (Cxcl13), transforming growth factor, beta 1 (Tgfb1), and interleukin 17 (Il17), while significant upregulation was observed around PEGDA hydrogels. Collectively, these findings strongly suggest that ZIP hydrogels exhibit superior biocompatibility and induce less pro‐fibrotic inflammation compared to PEGDA hydrogels when evaluated in a murine model.

### 2.5 ZIP Hydrogels Resist Long‐Term FBR in Mice

**Figure 5:**
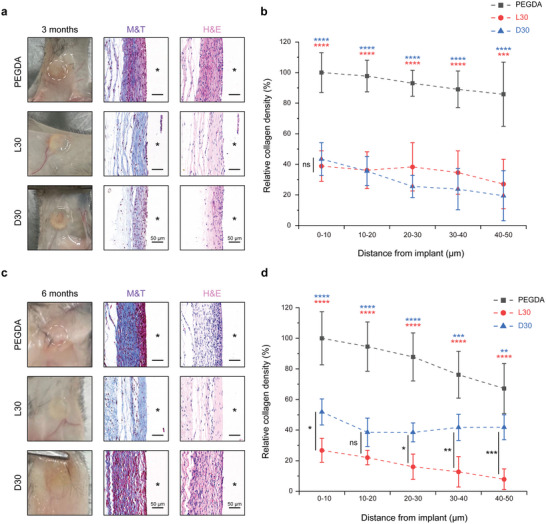
Long‐term anti‐fibrotic efficacy of ZIP hydrogels in mice. a–d) Digital photos, H&E‐, and M&T‐stained histological sections of excised 3‐month (a) or 6‐month (c) postimplantation. The white dashed circle represents the PEGDA and D30 disc that is not visible to the naked eye due to strong collagen capsule encapsulation after implantation. Asterisks in the section images denote the original locations of the implants. The density of the collagen capsules implanted for 3‐month (b) and 6‐month (d) post‐implantation is displayed (*n* = 6 mice per group, mean ± s.d.). Statistical significance was determined by one‐way analysis of variance (ANOVA) with Turkey post‐test. ^*^ *p* < 0.05, ^**^ *p* < 0.01, ^***^ *p* < 0.001, ^****^ *p* < 0.0001, ns: not significant.

The sustained preservation of biomaterials' resistance against fibrous capsule formation is imperative for ensuring the proper functionality of implantable devices in vivo. Therefore, we conducted extended implantation assessments involving the retrieval of hydrogels at both three and six months, followed by a fibrosis analysis (**Figure** **5**a–d). Notably, all examined ZIP hydrogel samples retained defined contours, displaying loosely arranged collagen matrix three‐ months post‐implantation, as evidenced by digital images and M&T staining. Nonetheless, the resistance of L30 hydrogels against fibrotic capsule formation remained robust even at the six‐month interval, while the boundaries of D30 hydrogels appeared blurred, coupled with an observable elevation in fibrous capsule density. In marked contrast, control PEGDA hydrogels exhibited severe fibrosis and were concealed under a dense collagen layer that had completely vanished by the three‐month period. Quantitative analysis of capsule density revealed a ∼25% and ∼50% collagen density for L30 and D30 gels, respectively, in comparison to PEGDA gels until six months post‐implantation. Compared with previously reported zwitterionic hydrogel implants, ZIP hydrogels demonstrated comparable or lower levels of fibrosis after long‐term implantation.^[^ ^18^, ^19^ ^]^ These findings underline the excellent FBR resistance and long‐term stability of ZIP hydrogels.

### 2.6 Antifibrotic Efficacy of ZIP Hydrogels in NHPs

**Figure 6:**
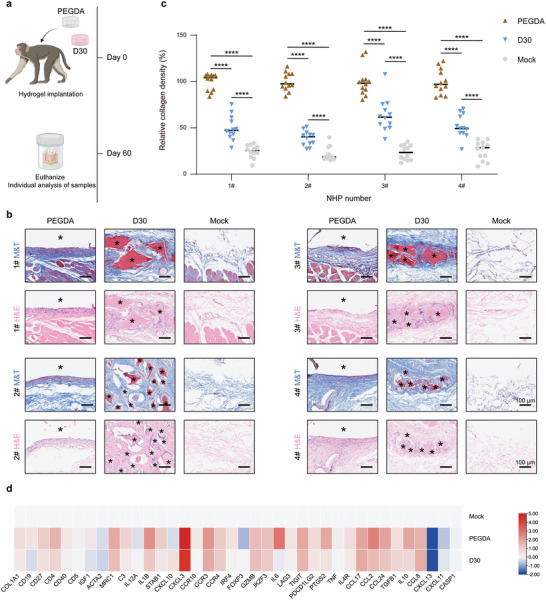
Long‐term antifibrotic efficacy of ZIP hydrogels in NHPs. a) Schematic depicting the cynomolgus monkeys study design. Figure created with BioRender. b) Digital photos, H&E‐, and Masson's trichrome (M&T)‐stained histological sections of excised SC tissue 2 months postimplant. Asterisks denote the original location of implants. c) Quantified data of collagen density of M&T staining in each monkey. d) Gene expression analysis of phenotypic markers in tissues surrounding the D30 and PEGDA materials 2 months post‐transplantation, with data normalized to the mock group and presented on a log_2_ scale (*n* = 3 monkeys per group). Statistical significance was determined by one‐way analysis of variance (ANOVA) with Turkey post‐test. ^*^ *p* < 0.05, ^**^ *p* < 0.01, ^***^ *p* < 0.001, ^****^ *p* < 0.0001, ns: not significant.

Next, our objective was to ascertain whether these discoveries could be extrapolated to a species of higher order. With this goal, we subcutaneously implanted both D30 and PEGDA hydrogels in healthy NHPs and retrieved the hydrogels after 2 months (**Figure** **6**a). Masson sections of PEGDA hydrogels implanted in NHPs showed a dense fibrotic capsule around the implants, whereas the D30 hydrogel was encapsulated by relatively loose collagen. The analysis of collagen density revealed that the collagen density near D30 hydrogels was ∼45%–60% of that of PEGDA hydrogels. These findings indicate that ZIP hydrogels exhibit a remarkable ability to alleviate fibrous tissue reactions within the NHP model (Figure 6b,c). Lastly, we conducted an RNA‐seq analysis to characterize the host‐mediated immune response following the subcutaneous implantation of D30 and PEGDA 2 months post‐implantation (Figure 6d). The results have revealed a significant difference in gene expression between D30 and PEGDA hydrogels. Notably, D30 hydrogels exhibited lower FBR‐associated gene expression, including the fibrosis marker COL1A1 and ACTA2; pro‐inflammatory cytokine IL1B and IL6; monocyte and macrophage chemoattractants C‐C motif chemokine ligand 2 (CCL2); T cell chemoattractants C‐C motif chemokine ligand 24 (CCL24); and markers for adaptive immune B cells and T cells, CD19 and CD4, in comparison to PEGDA implants. Our observations from this NHP study suggest that ZIP hydrogels demonstrate a significant reduction in the secretion of pro‐fibrotic factors and pro‐inflammatory cytokines, thereby eliciting a host response that is notably less pronounced in comparison to the response triggered by PEGDA implants.

## 3 Conclusion 

In summary, we have engineered a series of zwitterionic‐peptide (ZIP) hydrogel with adjustable enzymatic degradation characteristics and long‐term resistance against fibrotic capsule formation following implantation. The ZIP hydrogel exhibits exceptional anti‐fouling attributes, as demonstrated through in vitro assays involving protein, cells, and bacteria. Through the manipulation of the crosslinking degree and incorporation of D‐type amino acids, we have succeeded in tuning the in vivo stability of the hydrogel. When implanted in mice, the ZIP hydrogels induced minimal inflammatory response within 2 weeks while effectively inhibiting collagen capsule development for a duration of at least 6 months in mice and 2 months in NHPs. The expression levels of inflammatory factors and genes in the tissues surrounding ZIP hydrogels were comparable to the mock group without implantation, indicating the hydrogel's low fibrous capsule formation potential. Conversely, tissues surrounding PEGDA hydrogels exhibited elevated cytokine expression and upregulation of pro‐inflammatory genes. Recent studies have indicated that cells related to adaptive immunity also play a crucial role in the FBR process.^[^ ^47^, ^48^ ^]^ In this study, we did not investigate how the implantation of ZIP hydrogels affects the cells related to adaptive immunity (such as T cells). Future research could explore this aspect to better understand the specific anti‐fibrotic mechanism of the ZIP hydrogel. The combination of the pronounced anti‐fibrotic property and modifiable enzymatic degradation behavior positions ZIP hydrogels as a highly promising option for diverse applications in the realm of implantable biomaterials and biomedical devices.

## 4 Experimental Methods

### Materials

*N* ^ε^‐benzyloxycarbonyl‐α‐*tert*‐butyl‐_L_‐lysine hydrochloride, *N* ^ε^‐benzyloxycarbonyl‐α‐*tert*‐butyl‐_D_‐lysine hydrochloride, *δ*‐benzyl‐*N*‐*tert*‐butoxycarbonyl‐_L_‐glutamic acid, and *δ*‐benzyl‐*N*‐*tert*‐butoxycarbonyl‐_D_‐glutamic acid were supplied by Shanghai Hanhong Chemical Co., Ltd. 1‐(3‐Dimethylaminopropyl)−3‐ethylcarbodiimide hydrochloride (EDCl), 2‐hydroxybenzotriazole (HOBt), trifluoroacetic acid (TFA), anhydrous dimethylformamide (DMF), 33 wt% HBr/HOAc solution, *1H,1H,2H,2H*‐Perfluorooctyl‐trichlorosilane, *n*‐hexadecane, fluorescein isothiocyanate (FITC), Ammonium persulfate (APS), *N,N,N′,N′*‐Tetramethyl ethylenediamine (TEMED), and triethylamine (TEA) were supplied by Aladdin Reagent Co., Ltd. *o*‐Phenylenediamine (OPD) and Fluorescamine was purchased from Macklin Reagent Co., Ltd. Phosphate buffer solution (PBS) was supplied by Sangon Biotech Co., Ltd. Trypsin (Catalog. CR25200) was purchased from Cienry Co., Ltd. Fibrinogen and 4‐Morpholineethanesulfonic acid (MES) was purchased from Sigma‐Aldrich. Poly(ethyleneglycol) diacrylate (PEGDA), Mn = 2,000 Da was obtained from JenKem. The Silhouette infusion set (Silhouette Paradigm MMT‐381) was purchased from GEMCO Medical.

### Synthesis and Characterization of EK Polypeptides

The EK peptide was synthesized based on a previous work.^[^ ^26^ ^]^ The molecular weight of EK peptide was tested by GPC system (Waters 2515). The chemical structures of the EK peptide were confirmed by ^1^H NMR (400 MHz, D_2_O).

### Preparation of ZIP Hydrogels

The glass slide mold was fluorinated to facilitate demolding. The slide was immersed in 50 mL of hexadecane solution containing 500 µL 1H,1H,2H,2H‐perfluorooctyl‐trichlorosilane for 15 min under the nitrogen atmosphere. After the reaction, the slides were washed with acetone and ethanol and dried with nitrogen flow.

For the preparation of highly crosslinked ZIP hydrogels (L30 and D30), EK peptides (L‐type and D‐type) were dissolved in 0.1 M MES buffer (100 mg/mL, pH 6) followed by the addition of the crosslinking agent EDC·HCl (15% mole ratio of the amino group or carboxyl group of EK peptide). After vortex shaking for 10 s, the solution was placed at room temperature for 5 min, and then add the same mass of EDC·HCl as above. After the vortex, injected the solution into the mold immediately and reacted for 24 h. The resulting hydrogels were soaked in PBS (pH 7.4) for 48 h to remove the residual small molecules. The low crosslinked EK peptide hydrogels (L15, D15, and LD15) were prepared in a similar way. The total amount of EDC·HCl added was a 15% mole ratio of the amino or carboxyl group of EK peptide, added three times. PEGDA hydrogels were synthesized as a control. In brief, 60 mg PEGDA was dissolved in 1 mL DI water and ultrasound to dissolve completely. Then followed by the addition of 4 mg APS and 4 µL TEMED. After the vortex, injected the solution into the mold immediately and reacted for 24 h. The resulting hydrogels were soaked in PBS (pH 7.4) for 48 h to remove the residual small molecules.

### Crosslinking Degree test of ZIP Hydrogel

The crosslinking degrees of ZIP hydrogel were calculated by the number of residual amino groups. The determination of the remaining amino groups was achieved by using a fluorescamine assay. Briefly, ZIP hydrogels reached equilibrium in PBS 10 times to remove excessive small molecules. Each hydrogel was immersed in 750 µL DI water and 250 µL of fluorescamine solution (dimethyl sulfoxide, 3 mg mL^−1^). A standard curve was generated from the known EK peptide solution, spanning a concentration range from 0 to 0.1 mg mL^−1^. Following a 1 min incubation period at room temperature, the fluorescence intensity was quantified through excitation/emission measurements at 360 nm and 460 nm, respectively, using a microplate reader (Biotek Synergy H1, USA). Average data were acquired by testing four specimens for each sample.

### Water Content Test of ZIP Hydrogel

The as‐prepared hydrogels were immersed in PBS buffer until their mass did not change. The gels were then freeze‐dried to constant weights. The solid content of the hydrogels was calculated using the following equation:

(1) $$\mathrm{Solid}\;\mathrm{content}\;\left(\%\right)=\frac{m_{dry}}{m_{swollen}}\times 100\%$$

where m_dry_ and m_swollen_ are the masses of the hydrogels at fully dried and fully swollen states, respectively.

### Rheological Analysis

The rheological tests of ZIP hydrogels with different EDCl feeding ratios and amino configurations were performed on a DHR‐2 rheometer (TA instruments) equipped with a 25 mm‐diameter parallel plate. All tests were performed at 25°C. The strain amplitude sweep tests were performed with shear strain increasing from 0.005% to 1100%.

### Compressive Test

Hydrogel specimens were fabricated as 8‐mm diameter and 8‐mm thick cylindrical shapes for subsequent compressive testing. The compression analysis of these hydrogels was conducted using a universal testing machine (Instron 5982) equipped with a 50 N load cell, all conducted at room temperature. The testing protocol involved setting a crosshead speed of 2 mm min^−1^, and a compressive strain limit of 90% was established to safeguard the equipment. Average data were acquired by testing four specimens for each sample.

### Fibrinogen Fouling Test

The protein adsorption on ZIP hydrogels was conducted by enzyme‐linked immunosorbent assay (ELISA). Fibrinogen (Fg) was selected as the test protein to examine the anti‐fouling properties of the ZIP hydrogel samples (L30 and D30). In brief, hydrogel samples were prepared by cutting 1 mm‐thick hydrogel sheets into 8 mm diameter discs using a biopuncher. In addition, 1mm‐thick PEGDA hydrogel sheets were cut into 8 mm diameter discs using a biopuncher which were used as controls. The discs were initially situated in a 24‐well plate and subjected to a 5‐min equilibration period in PBS. Next, the samples were immersed in 1 mL of 1 mg mL^−1^ Fg in PBS buffer for a duration of 1 h, followed by three thorough washes with PBS buffer. The samples were then relocated to fresh wells and exposed to 1 mL of horseradish peroxidase (HRP) conjugated anti‐fibrinogen antibody (1 µg mL^−1^) from Bellancom Chemistry (catalog no. G11390) in PBS buffer for 0.5 h. All samples were then transferred to new wells after another five washes with pure PBS buffer. Subsequently, 1 mL of *o*‐phenylenediamine (OPD) chromogen solution was introduced. Following a 15‐min incubation period, the enzymatic reaction was stopped by the addition of an equal volume of 1N hydrochloric acid (HCl). The absorbance reading at 490 nm was then recorded using a microplate reader (MODEL 550, Bio‐Rad), and was normalized to that of the PEGDA sample. Average data were acquired from six specimens.

### Cell Adhesion Assay

Human primary umbilical vein endothelial cells (ECs) were freshly isolated from human umbilical veins and arteries following approval from the local medical ethics committee. These ECs were cultured in an EC medium, which was supplemented with 10% fetal bovine serum (FBS), 20 mg mL^−1^ of endothelial cell growth supplement, 60 µg mL^−1^ of penicillin, and 100 µg mL^−1^ of streptomycin in a culture dish at 37 °C under a humidified atmosphere containing 5% CO_2_. ZIP hydrogel samples (L30 and D30) were cut from the 1 mm thick hydrogel sheet into 8 mm diameter discs with a biopuncher. TCPS and PEGDA hydrogel were used as controls. For fluorescent imaging, ECs were stained by the addition of CellTracker Orange CMTMR (5‐(and‐6)‐(((4‐ chloromethyl)benzoyl)amino)tetramethylrhodamine) (1 µg mL^−1^) from Thermo Fisher (catalog no. C2927). This staining process was carried out for 30 min prior to trypsinization. Subsequently, the EC suspensions in EC medium, supplemented with 20 mg mL^−1^ ECGS and 1% UI mL^−1^ streptomycin−penicillin, were seeded onto sample surfaces in 48‐well plates at a cell density of 10000 cells cm^−2^. The cells were then incubated for 4 h. Following incubation, the hydrogel was gently rinsed with sterile PBS to eliminate non‐adherent cells, and fluorescence images were captured using a fluorescence microscope (Nikon DS‐Ri2). For quantifications, the samples were gently transferred to new wells, followed by adding 200 µL fresh medium containing 20 µL CCK‐8 and incubated for another 4 h. The cell viability was ultimately assessed using a Bio‐Rad microplate reader, measuring absorbance at both 450 and 570 nm wavelengths (as calibration wavelength). Absorbance was normalized using cells cultured on TCPS sample as 100%. Average data were acquired from four specimens.

### Bacteria Adhesion Analysis

A gram‐positive strain of MRSA and a gram‐negative strain of *P. aeruginosa* were cultivated separately in Luria‐Bertani (LB) medium for 24 h at 37 °C with continuous shaking at 200 rpm. The suspended cultures were diluted and further incubated in LB for 2 h to reach the exponential growth phase. Once the second suspended culture achieved an optical density of 1.0 at 600 nm, the bacteria were centrifuged at 5,000 rpm, resuspended in sterile PBS to a concentration of approximately 1 × 10^8^ CFU mL^−1^, and stained with 0.5 µm SYTO9 for 20 min. Exponentially grown bacteria were subsequently applied to the hydrogel samples within the wells and cultured for 24 h at 37°C. Following the incubation period, the wells were gently washed with sterile PBS to eliminate non‐adherent bacteria, and the outcomes were directly observed using Nikon Eclipse Ts2.

### In Vitro Degradation Analysis

ZIP hydrogel samples (L‐type, D‐type, and LD‐type) with different crosslinking degrees were cut from the 1 mm thick hydrogel sheet into 8 mm diameter discs with a biopuncher. For fluorescent imaging and quantification, samples were immersed in glass bottles filled with 5 µg mL^−1^ FITC solution in PBS. After 24 h incubation, hydrogel samples were washed with PBS five times to remove unreacted FITC. Afterward, submerge the samples in 3 mL trypsin solution (50 U mL^−1^ in PBS) and all these bottles were incubated at 37°C and shaken at a speed of 110 rpm in the dark for 24 h. The fluorescence images were taken before and after 24 h trypsin treatment. The fluorescence intensity of the solution after enzymatic treatment at different times was recorded by a microplate reader (MODEL 550, Bio‐Rad) (excitation and emission wavelength at 490 nm and 520 nm, respectively). Average data were acquired from three specimens.

### In Vivo Degradation Analysis

High‐frequency ultrasound Imaging (Vinno 6 Vet; 23–97 MHz) was employed to assess gel degradation in vivo. Axial images were captured to visualize both the skin and the hydrogel. In the case of ZIP hydrogels with high crosslinking degrees, imaging was conducted at two time points: 1 week and 16 weeks post‐implantation. For gels with low crosslinking degrees, imaging was conducted at four time points: 1, 2, 4, and 8 weeks post‐implantation. To further corroborate the degradation process, photographs of the gel beneath the skin were taken at each of these time points. Average data were collected from three specimens.

### Animal Work

Mice experiments were in compliance with the relevant regulations, and all protocols were approved by the Institutional Animal Care and Use Committee, Zhejiang Academy of Medical Sciences (Approval number: ZJCLA‐IACUC‐20010235). Wild‐type (WT) female C57BL/6 mice of 6–8 weeks of age were sourced from the animal center at Zhejiang Academy of Medical Sciences. These mice were provided with a standard laboratory diet and subjected to a 12‐h light/12‐h dark cycle.

For procedures in mice, the ZIP hydrogel (L15, D15, L30, and D30) and PEGDA hydrogel sheets were cut into discs with a biophysical punch (4 mm in diameter). The hydrogel samples were sterilized using 30 min of UV irradiation and were then subcutaneously implanted in C57BL/6 female mice. The implantation procedure was as follows. In brief, mice were anesthetized with 3% isoflurane in oxygen, shaved, and disinfected the skin with iodine. An approximately 8 mm longitudinal incision was made on the dorsal surface, and subcutaneous pockets were created using blunt forceps, about 0.5 cm away from the incision, for the implantation of the hydrogel discs. After implantation, the incisions were closed using 5‐0 taper‐tipped PGA absorbable sutures. The mice were monitored until they had fully recovered from anesthesia and were raised for varying durations: 1 week, 2 weeks, 4 weeks, 8 weeks, 3 months, 4 months, and 6 months, respectively. The mice exhibited normal growth with no signs of discomfort following implantation, and no changes in body weight were observed throughout the entire experimental period.

NHPs experiments were in compliance with the relevant regulations, and all protocols were approved by the Laboratory Animal Ethics Committee of the Second Affiliated Hospital, School of Medicine, Zhejiang University (Approval number: No. 30 of 2023). Male cynomolgus monkeys, aged 2 to 3 years, with body weight ranging from 2 to 3.5 kg, were sourced from Suzhou Xishan Zhongke Laboratory Animal Co. Ltd., which has been certified by the Association for Assessment and Accreditation of Laboratory Animal Care. The monkeys were provided with a standard laboratory diet and adhered to a 12‐h light/12‐h dark cycle for their daily routines.

For procedures in NHPs, the ZIP hydrogel (D30) and PEGDA hydrogel discs were sterilized with an electron beam (10 kGy), and implanted subcutaneously in cynomolgus monkeys. The implantation procedure was as follows. To prepare the animals for the implantation procedure, they were anesthetized by way of intramuscular injection, with ketamine administered at a rate of 5 mg k^−1^g (Gutian Pharmaceutical) and midazolam at 0.2 mg k^−1 ^g (Ehwa Pharma). Subsequently, the animals were connected to an animal ventilator (Matrx, USA) and ventilated with room air. To ensure their comfort and maintain body temperature, the animals were placed on circulating warm water‐based blankets and kept covered throughout the entire procedure. The implantation process involved making approximately 1 cm dorsal skin incisions on the left and right lateral sides of the thoracic spine. Subcutaneous pockets, extending ventrally to a depth of about 5 cm, were created using blunt dissection. In the left and right subcutaneous pockets, D30 and PEGDA discs were respectively placed. Following implantation, the incisions were meticulously closed using 5‐0 taper‐tipped PGA absorbable sutures. For postsurgical pain management, each animal received a single injection of 50000 U kg^−1^ perioperative penicillin G benzathine/penicillin G procaine (Combi‐Pen) and subcutaneous administration of meloxicam. Meloxicam was administered at a rate of 0.2 mg k^−1^g on the first day and 0.1 mg k^−1^g on the subsequent two days.

### In Vivo Insulin Infusion Catheter Implantation in Mice

Healthy C57BL/6 mice were anesthetized with 3% isoflurane in oxygen, shaved, and disinfected the skin with iodine. An approximately 8 mm longitudinal incision was made on the dorsal surface, and subcutaneous pockets were created using blunt forceps, about 0.5 cm away from the incision. For the untreated group, an adequate pocket was created and then the sterile insulin infusion catheter (disassembled from a commercial Silhouette infusion set) was implanted into the SC space such that the catheter lay parallel to the dorsal midline. For the treated group, the prepared ZIP gel (∼100 µL) was injected at the distal end of the SC pocket first and the catheter was then implanted into the SC space to ensure that the tip end was enclosed by the zwitterionic gel. After implantation, the incisions were closed using 5‐0 taper‐tipped PGA absorbable sutures. The mice were monitored until they had fully recovered from anesthesia and were raised for 2 weeks. The mice exhibited normal growth with no signs of discomfort following implantation, and no changes in body weight were observed throughout the entire experimental period.

### Retrieval of Tissues and Hydrogels

For the mouse experiments, at various time intervals, including 1 week, 2 weeks, 4 weeks, 8 weeks, 3 months, 4 months, and 6 months, respectively. Mice were sacrificed and the hydrogel samples along with the surrounding tissues, were excised and collected. These explanted samples were then either fixed in a 10% formaldehyde solution (for use in histology) or flash‐frozen (for RNA analysis).

In the case of NHP SubQ retrievals, the process began with the preparation of animals for live excision procedures, following the same protocol as the initial implantation procedure (as previously described). At the two‐month retrieval time points, 8 mm biopsy punches were utilized to sample both the skin and the subcutaneous space. After the retrieval, the sites were carefully closed using 5‐0 taper‐tipped PGA absorbable sutures.

### Immunostaining of Tissue Sections, Microscopy, and Quantitative Image Analysis

Tissue sections from paraformaldehyde‐fixed samples were processed for immunostaining and microscopy. The sections, ranging from 3 to 5 µm in thickness, were cut after dehydration and paraffin embedding, followed by deparaffinization and rehydration. Staining involved hematoxylin and eosin for cellularity assessment and Masson's trichrome for collagen examination using standard histological procedures. For immunohistochemistry, sections underwent heat‐induced epitope retrieval in a 0.1 M citrate buffer at pH 6, conducted for 15 min at 95 °C, and were subsequently rehydrated through three washes with a PBS solution containing 0.1% Tween wash buffer. Immunohistochemical staining followed the manufacturer's guidelines, utilizing the Diaminobenzidine (DAB) chromogenic reagent Kit (DAKO; catalog no. K5007). In summary, the sections were initially blocked with a peroxidase blocking solution for 10 min at room temperature, followed by three washes in a PBS solution with 0.1% Tween wash buffer. Primary antibodies specific to CCR‐7 (rabbit IgG; dilution 1:500; Abcam; catalog no. ab253187), TNF‐α (goat IgG; dilution 15 µg mL^−1^; R&D systems; catalog no. AF‐410), CD31 (Goat IgG; dilution 15 µg mL^−1^; R&D systems; catalog no. AF‐3628), and IL‐6 (goat IgG; dilution 15 µg mL^−1^; R&D systems; catalog no. AF‐406) were used. This step was followed by incubation with anti‐rabbit or anti‐goat secondary horseradish peroxidase‐conjugated antibodies: goat anti‐rabbit IgG (HRP polymer; SE134; Solarbio) and rabbit anti‐goat IgG (HRP polymer; GB23204; Servicebio)). For immunofluorescence staining, tissue sections were subjected to incubation in 0.1 M citrate buffer (pH 6) for 15 min at 95 °C, followed by rehydration through three washes in a wash buffer composed of TBS + 0.025% Triton X‐100. These sections were then immersed in 10% goat serum for 60 min and subsequently underwent overnight incubation with primary antibodies directed against F4/80 (rabbit IgG; dilution 1:5,000; Abcam; catalog no. ab300421). The isotype‐specific secondary antibody Alexa Fluor 488‐conjugated goat anti‐rabbit IgG (H + L) F(ab')_2_ Fragment (dilution 1:200; catalog no. 4412S; Cell Signaling Technology) was used to detect primary antibodies. 4′,6‐diamidino‐ 2‐phenylindole (DAPI) dihydrochloride (1:50; D9542; Sigma–Aldrich) was used to visualize the nuclei. Images were acquired with a microscope (Nikon intensilight CHGFI) equipped with the NIS‐Elements AR software and a Virtual Slide Microscope (VS120‐S6‐W, Olympus, Japan). Collagen density was determined by measuring the percentage of blue‐pixel coverage in the M&T images of tissues within 50 µm intervals (at 10 µm steps) from the material‐tissue interface. Regions positive (optical density value) for TNF‐α, IL‐6, and CCR‐7 were calculated as a percentage of the positive staining within the entire deep or fascia‐facing capsule area using ImageJ. All images were processed using Adobe Photoshop 2023 (Adobe Systems), with consistent enhancements to contrast and brightness applied to all representative images utilized.

### Protein Extraction

Proteins were extracted from skin tissue surrounding implant L30, PEGDA, and from non‐implanted tissue for subsequent characterization. In brief, RIPA lysis buffer (containing phenylmethylsulfonyl fluoride) from Solarbio (catalog no. R0010) was added to the isolated skin tissue, then the tissue was thoroughly triturated with a homogenizer and lysed for 30 min on ice. After sufficient lysis, the tissue lysate was centrifuged at 10,000 g for 5 min at 4°C. Then the supernatant was transferred to a new tube. The extracted protein was quantified using a BCA kit from Beyotime (catalog no. P0010S), and then the protein was aliquoted and stored at −80°C.

### Cytokine Profiling Analysis

For cytokine profiling analysis, a Proteome Profiler antibody array from R&D Systems (catalog no. ARY006) was employed. Tissue samples collected after 2 weeks of implantation were compared to a mock group without implantation, serving as the control. Proteins from the tissue samples were extracted. Subsequently, 2 mL of blocking buffer was dispensed into each well of a 4‐well multi‐dish containing an antibody array membrane and allowed to incubate for 1 h on a rocking platform shaker. Following aspiration of the blocking buffer, 1.5 mL of sample solutions, each containing 300 µg of proteins and 15 µL of detection antibody cocktail, were added to each well and incubated overnight at 2–8°C on a shaker. Each membrane was then placed into individual plastic containers and washed three times using a wash buffer. The membrane was returned to the four‐well multidish, which contained 2 mL of diluted Streptavidin‐HRP (at a 1:2,000 ratio), and was incubated for 30 min on a shaker. After three additional washes with the wash buffer, the membranes were incubated with 1 mL of Chemi Reagent Mix for 1 min and subsequently placed in an autoradiography film cassette (ChemiScope 6000, Clinx) to capture chemiluminescence. The gray value of the results was measured using ImageJ software.

### RNA‐seq and Data Analysis

For RNA‐seq and data analysis, all equipment used underwent treatment with diethyl pyrocarbonate (DEPC) to ensure RNA‐DNase‐free conditions before the experiment. Following the implantation of hydrogels, tissue samples adjacent to the implant materials were excised and immediately immersed in RNA‐later solution obtained from Sigma‐Aldrich (catalog no. R0901) to preserve the samples, with a mock group that did not undergo implantation serving as the control. RNA samples were assessed and quantified using a NanoDrop and Agilent 2100 bioanalyzer (Thermo Fisher Scientific, MA, USA). RNA sequencing libraries were then constructed and sequenced on the BGISEQ‐500 platforms. Data analysis included identifying significant differences between the PEGDA and L30 group data, with a *p* value threshold of less than 0.05.

### Quantitative PCR Assay

Total RNA was extracted from skin tissue near the implants by using TRIzol reagent and RNase‐Free DNase Set (Qiagen). qRT‐PCR was conducted by using Power SYBR Green Master Mix (Applied Biosystems) according to the manufacturer's instructions. The extracted and purified RNA samples (500 ng) were reverse transcribed into complementary DNA (cDNA) using a SuperScript III First‐Strand Synthesis SuperMix (Thermo Fisher). The qRT‐PCRs were performed on the Real‐Time PCR Detection Systems (CFX384, Bio‐Rad, USA) using the manufacturer's recommended settings for quantitative and relative expression. Primers used for qRT‐PCR are listed in Table S4 (Supporting Information).

### Statistical Analysis

Details of the sample size and appropriate statistical test were included in the figure captions. The data were expressed as means ± s.d. of technical replicates. The data were analyzed for statistical significance by unpaired, two‐tailed t‐test and one‐way analysis of variance (ANOVA) with Turkey post‐test using SPSS Statistics 26.0.

## Conflict of Interest

The authors declare no conflict of interest.

## Supporting information

Supporting Information

## Data Availability

The data that support the findings of this study are available from the corresponding author upon reasonable request.
