# Supplementary material for: Poly(Glutamic Acid‐Lysine) Hydrogels with Alternating Sequence Resist the Foreign Body Response in Rodents and Non‐Human Primates
Source: Adv Sci (Weinh). 2024 Feb 25;11(16):2308077. doi: 10.1002/advs.202308077 (PMC11040334; doi:10.1002/advs.202308077)
Supplement: Supplementary file 1 — Supporting Information [file ADVS-11-2308077-s001.pdf]

ADVANCED SCIENCE
Open Access

Supporting Information

for *Adv. Sci.*, DOI 10.1002/advs.202308077

Poly(Glutamic Acid-Lysine) Hydrogels with Alternating Sequence Resist the Foreign Body Response in Rodents
and Non-Human Primates

Xianchi Zhou, Wenzhong Cao, Yongcheng Chen, Zihao Zhu, Yifeng Chen, Yanwen Ni, Zuolong Liu, Fan Jia, Zhouyu Lu, Yang Ye, Haijie Han, Ke Yao, Weifeng Liu, Xinyue Wei, Shengfu Chen, Youxiang Wang, Jian Ji and Peng Zhang**

Supporting Information

Poly(glutamic acid-lysine) Hydrogels with Alternating Sequence Resist the Foreign Body Response in Rodents and Non-human Primates.

Xianchi Zhou, Wenzhong Cao, Yongcheng Chen, Zihao Zhu, Yifeng Chen, Yanwen Ni, Zuolong Liu, Fan Jia, Zhouyu Lu, Yang Ye, Haijie Han, Ke Yao, Weifeng Liu, Xinyue Wei, Shengfu Chen, Youxiang Wang, Jian Ji, and Peng Zhang**

This PDF file includes:

Supporting text

Figures S1 to S8

Tables S1 to S3

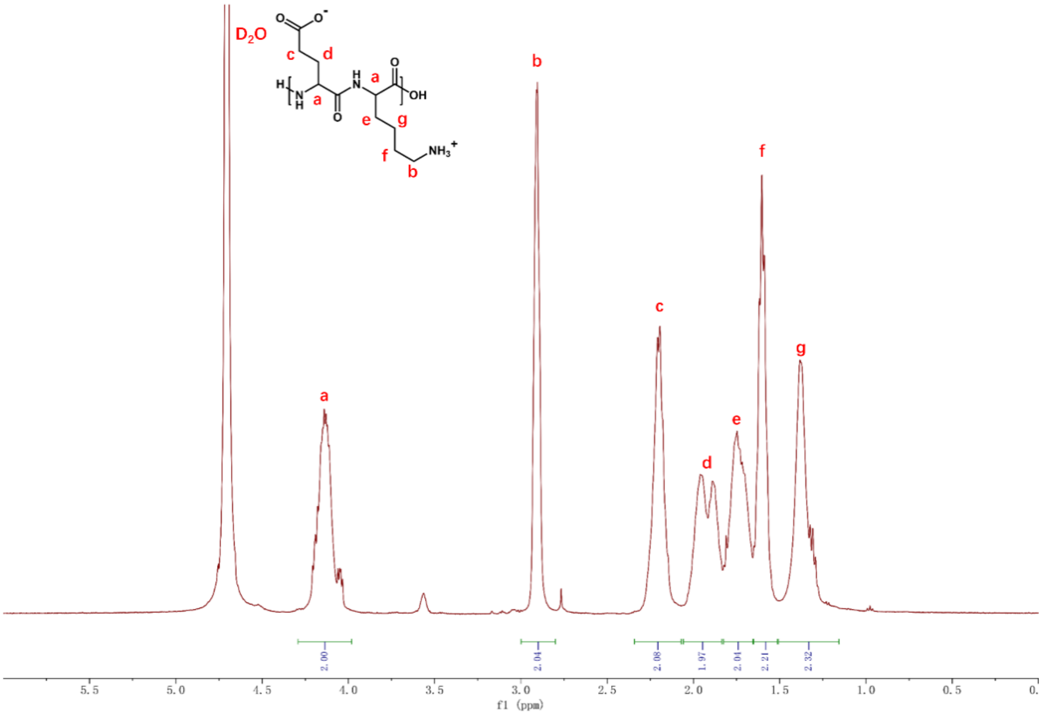

Figure S1. ^{1}H NMR (400 MHz, D_2O) spectrum of the EK polypeptide.

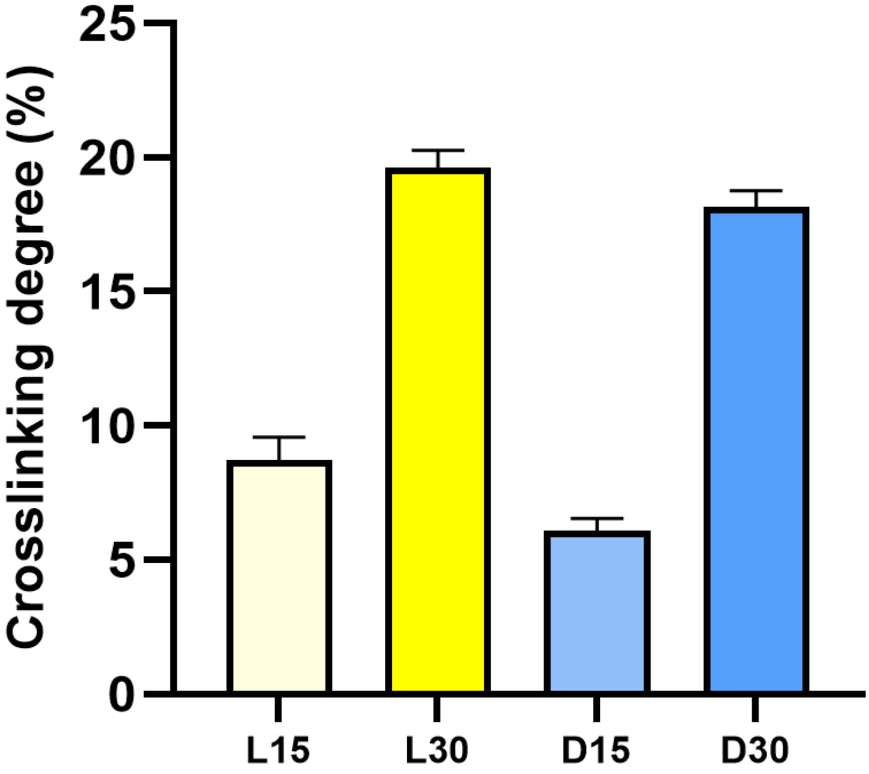

Figure S2. Crosslinking degree of the ZIP hydrogels. The crosslinking degree of the ZIP hydrogel was determined through fluorescamine assay. (n = 3, mean values ± s.d.).

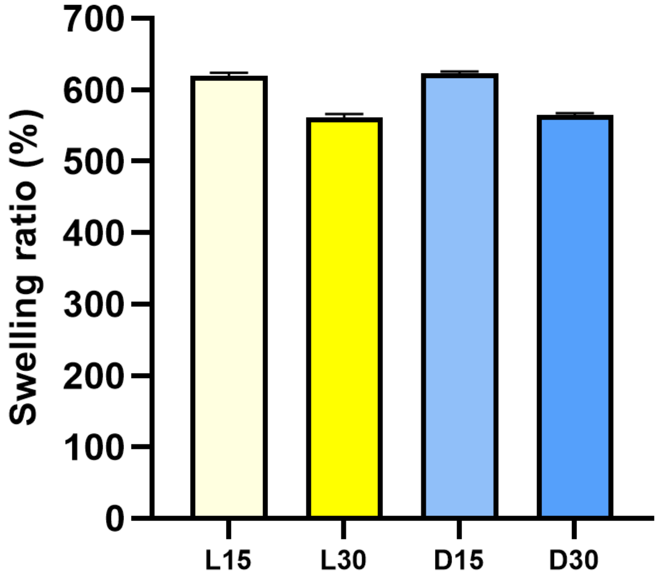

Figure S3. Swelling ratio of the ZIP hydrogels. (n = 3, mean values ± s.d.).

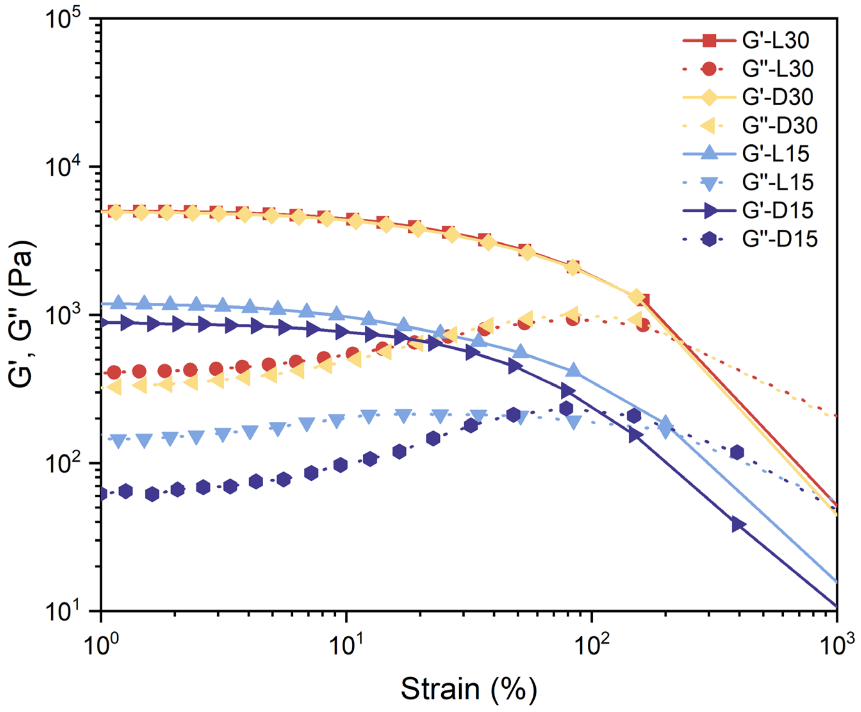

Figure S4. Rheological tests of the ZIP hydrogels. (n = 3).

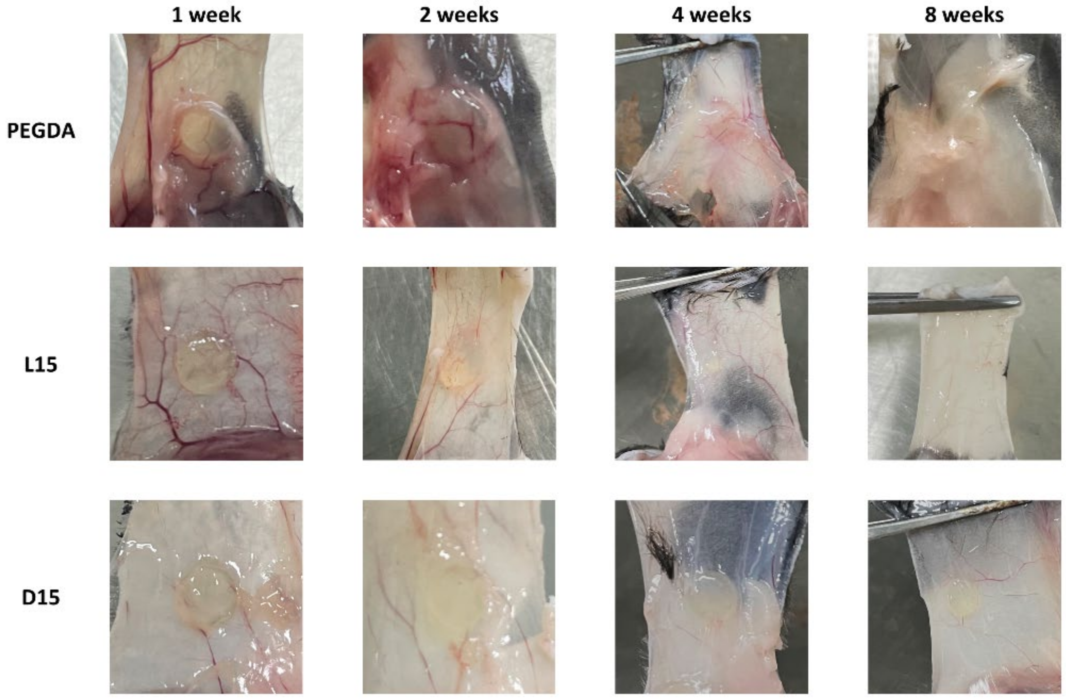

Figure S5. Digital photographs of the ZIP hydrogels at different time points after subcutaneous implantation. (n = 3).

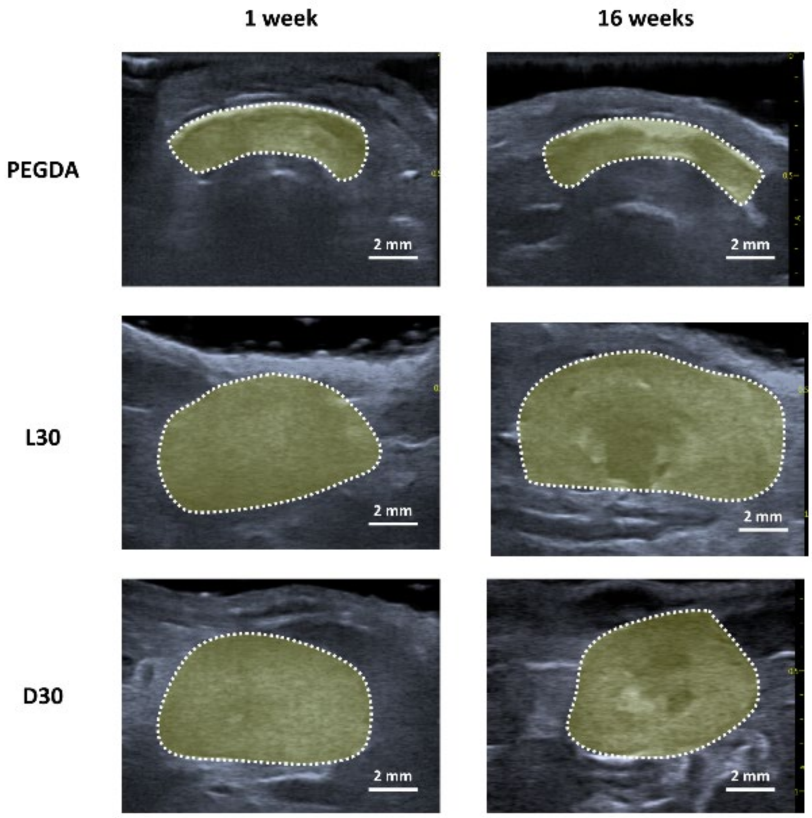

Figure S6. HFUS imaging evaluated changes in the ZIP hydrogels with high crosslinking degrees. (n = 3).

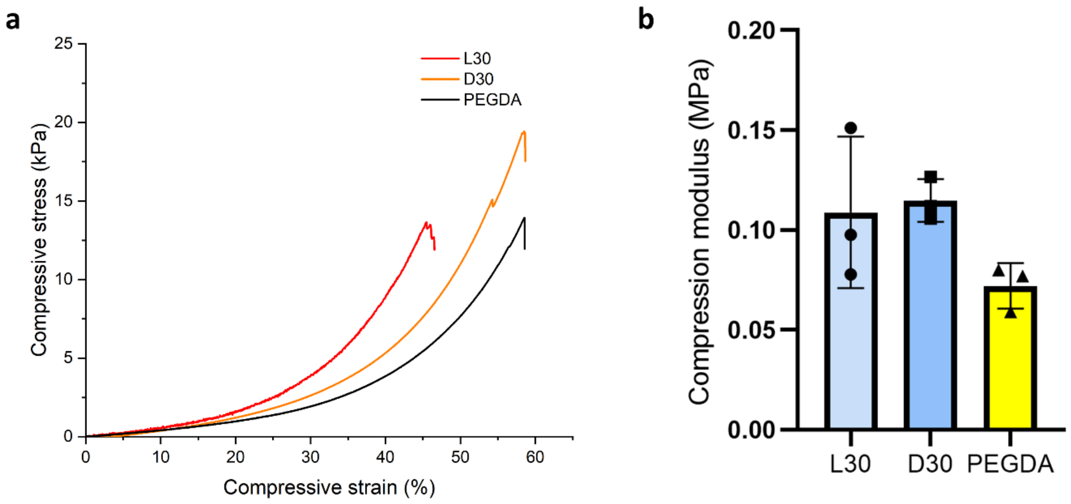

Figure S7. (a) Compressive curves of the hydrogels. (b) Compressive modulus of the hydrogels. (n = 3, mean values ± s.d.).

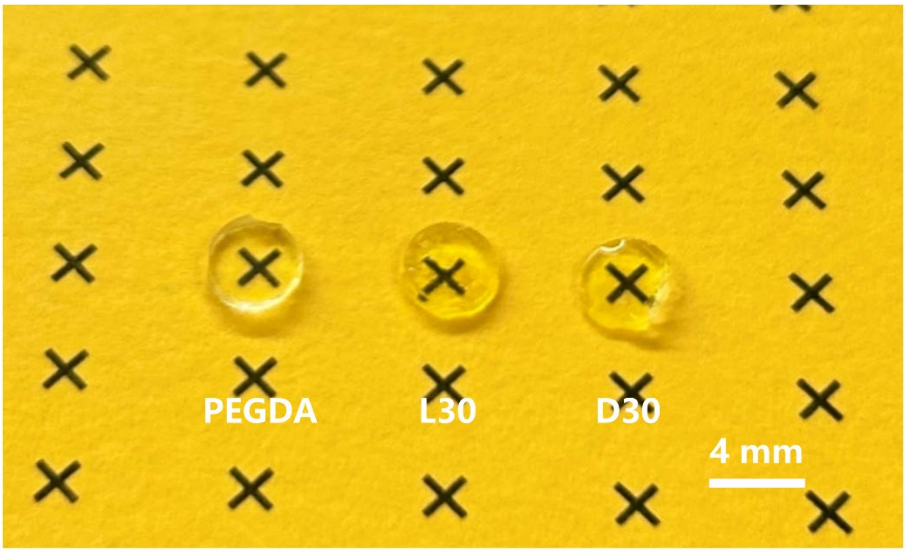

Figure S8. Appearance and size of the PEGDA and ZIP hydrogels for subsequent animal experiments. All implants have a thickness of 1mm.

Table S1. Characterization of the ZIP hydrogel precursor polymers.
[a] Đ means the dispersity that was measured by GPC.

	Mn (Da)	Mw (Da)	Đ[a]
EK peptide	14111	18200	1.29

Table S2. The components for the ZIP hydrogel preparation.

Hydrogel	Components
L15	10 wt% L-type EK polypeptide in MES buffer, with 15 mol% EDCI
D15	10 wt% D-type EK polypeptide in MES buffer, with 15 mol% EDCI
LD15	5 wt% L-type and 5 wt% D-type EK polypeptide in MES buffer, with 15 mol% EDCI
L30	10 wt% L-type EK polypeptide in MES buffer, with 30 mol% EDCI
D30	10 wt% D-type EK polypeptide in MES buffer, with 30 mol% EDCI

Table S3. The mouse cytokine array coordinates.

Coordinate	Target/Control	Alternate Nomenclature	Coordinate	Target/Control	Alternate Nomenclature
A1, A2	Reference Spot	——	C17, C18	IL-16	——
A23, A24	Reference Spot	——	C19, C20	IL-17	——
B1, B2	BLC	CXCL13/BCA-1	C21, C22	IL-23	——
B3, B4	C5/C5a	Complement Component 5a	C23, C24	IL-27	——
B5, B6	G-CSF	——	D1, D2	IP-10	CXCL10/CRG-2
B7, B8	GM-CSF	——	D3, D4	I-TAC	CXCL11
B9, B10	I-309	CCL1/TCA-3	D5, D6	KC	CXCL1
B11, B12	Eotaxin	CCL11	D7, D8	M-CSF	——
B13, B14	sICAM-1	CD54	D9, D10	JE	CCL2/MCP-1
B15, B16	IFN-γ	——	D11, D12	MCP-5	CCL12
B17, B18	IL-1α	IL-1F1	D13, D14	MIG	CXCL9
B19, B20	IL-1β	IL-1F2	D15, D16	MIP-1α	CCL3
B21, B22	IL-1ra	IL-1F3	D17, D18	MIP-1β	CCL4
B23, B24	IL-2	——	D19, D20	MIP-2	CXCL2
C1, C2	IL-3	——	D21, D22	RANTES	CCL5
C3, C4	IL-4	——	D23, D24	SDF-1	CXCL12
C5, C6	IL-5	——	E1, E2	TARC	CCL17
C7, C8	IL-6	——	E3, E4	TIMP-1	——
C9, C10	IL-7	——	E5, E6	TNF-α	——
C11, C12	IL-10	——	E7, E8	TREM-1	——
C13, C14	IL-13	——	F1, F2	Reference Spot	——
C15, C16	IL-12 p70	——	F23, F24	PBS(Negative Control)	Control(-)

Table S4. List of mouse mRNA primers for qPCR analysis.

Primer	Primer Sequences (5' to 3')
Col1a1	Forward: CCTTCTGGACCCGTTGGCAAAGAT
	Reverse: GGCTACCCTGAGAACCACGAACA
Il1b	Forward: GGCAGGCAGTATCACTCATTGTG
	Reverse: GCTCATGTCCTCATCCTGGAAG
Tnfa	Forward: GACCCTCACACTCAGATCATCTTCT
	Reverse: GCTACGACGTGGGCTACAG
Acta2	Forward: GCACCCAGCACCATGAAGATCAAG
	Reverse: GAAGGTAGACAGCGAAGCCAGGAT
Il17a	Forward: CACCGCAATGAAGACCCTGATA
	Reverse: CCAGGATCTCTTGCTGGATGAGA
